# Tract Sealing Techniques for Pneumothorax and Drainage Prevention After CT-Guided Lung Biopsy: A Systematic Review and Meta-Analysis

**DOI:** 10.3390/diagnostics16060824

**Published:** 2026-03-10

**Authors:** Andrei Roman, Nicoleta-Anca Lobonț-Terec, Roxana Pintican, Bogdan Fetica, Paul Kubelac, Zsolt Fekete, Alexandra Cristina Preda, Andrei Pașca, Călin Schiau, Csaba Csutak

**Affiliations:** 1 Radiology Department, Prof. Dr. Ion Chiricuţă Institute of Oncology, 400015 Cluj-Napoca, Romania; andrei.roman678@gmail.com (A.R.); roxanapintican@gmail.com (R.P.); 2 Radiology Department, Iuliu Hațieganu University of Medicine and Pharmacy, 400337 Cluj-Napoca, Romania; calin.schiau@yahoo.com (C.S.); csutakcsaba@yahoo.com (C.C.); 3 Faculty of Medicine, Iuliu Hațieganu University of Medicine and Pharmacy, 400337 Cluj-Napoca, Romania; 4 Pathology Department, Prof. Dr. Ion Chiricuţă Institute of Oncology, 400015 Cluj-Napoca, Romania; feticab@yahoo.com; 5 Pathology Department, Iuliu Hațieganu University of Medicine and Pharmacy, 400337 Cluj-Napoca, Romania; 6 Faculty of Medical and Health Sciences, Babeș-Bolyai University, 400084 Cluj-Napoca, Romania; paulkubelac@gmail.com; 7 Oncology Department, Prof. Dr. Ion Chiricuţă Institute of Oncology, 400015 Cluj-Napoca, Romania; preda.alexandra_cristina@yahoo.com; 8 Radiotherapy Department, Prof. Dr. Ion Chiricuţă Institute of Oncology, 400015 Cluj-Napoca, Romania; drfekete@gmail.com; 9 Radiotherapy Department, Iuliu Hațieganu University of Medicine and Pharmacy, 400337 Cluj-Napoca, Romania; 10 Oncology Department, Iuliu Hațieganu University of Medicine and Pharmacy, 400337 Cluj-Napoca, Romania; 11 Surgery Department, Prof. Dr. Ion Chiricuţă Institute of Oncology, 400015 Cluj-Napoca, Romania; pasca_andrei@elearn.umfcluj.ro; 12 Surgery Department, Iuliu Hațieganu University of Medicine and Pharmacy, 400337 Cluj-Napoca, Romania; 13 Radiology Department, Cluj-Napoca Emergency County Hospital, 400000 Cluj-Napoca, Romania

**Keywords:** CT-guided biopsy, lung, pneumothorax, drainage, tract sealing

## Abstract

**Background/Objectives**: Our goal was to evaluate the effectiveness of tract sealing agents in reducing pneumothorax and chest drainage insertion following CT-guided lung biopsy (CLB), and to assess the certainty of supporting evidence. **Methods**: A systematic review and meta-analysis were conducted according to PRISMA 2020 guidelines (PROSPERO: CRD42024608747). Four health science databases (ScienceDirect, PubMed, Scopus, and Cochrane Library) were searched up to 13 October 2025. Randomized controlled trials and cohort studies reporting tract sealing after CLB were included. Outcomes were post-procedural pneumothorax and pleural drainage insertion. Both were analyzed as dichotomous variables using random-effects meta-analysis with the Mantel–Haenszel method. Statistical heterogeneity was assessed using the I^2^ statistic. Results were considered statistically significant for *p* < 0.05. Study quality was assessed using the Revised Cochrane risk-of-bias tool for randomized trials (RoB 2) and the Risk Of Bias In Non-randomized Studies—of Interventions, Version 2 (ROBINS-I V2) tool for cohort studies. **Results**: A total of 3328 records were initially retrieved, with 37 studies (13,107 patients, 7161 male and 4526 female) meeting the inclusion criteria. Sealing agents included saline solution, hydrogel plug, gelatin sponge, autologous blood patch, saline + rapid roll-over, hemocoagulase, gelatin sponge + hemocoagulase, and fibrin glue. Meta-analysis demonstrated significant reductions in pneumothorax and drainage insertion with saline solution (pneumothorax: OR = 0.35; 95% CI 0.25–0.48; *p* < 0.00001; drainage: OR = 0.22, 95% CI 0.11–0.43; *p* < 0.00001), gelatin sponge (pneumothorax: OR = 0.44, 95% CI 0.37–0.53; *p* < 0.00001; drainage: OR = 0.40, 95% CI 0.29–0.54; *p* < 0.00001), autologous blood patch (pneumothorax: OR = 0.50, 95% CI 0.40–0.62; *p* < 0.00001; drainage: OR = 0.40, 95% CI 0.27–0.59; *p* < 0.00001), and hydrogel plug (pneumothorax: OR = 0.65, 95% CI 0.50–0.85; *p* = 0.001; drainage: OR = 0.44, 95% CI 0.25–0.76; *p* < 0.004). **Conclusions**: Saline solution, hydrogel plug, gelatin sponge, and autologous blood patch are sealing agents that are effective at lowering the risk of pneumothorax and drainage insertion following CLB.

## 1. Introduction

CT-guided lung biopsy (CLB) is widely used in the diagnosis of pulmonary tumors. Pneumothorax, defined as the presence of air within the pleural cavity, resulting in partial or complete lung collapse, is the most frequent complication of CLB, occurring in approximately 4.3–52.4% of procedures, with 0–15% requiring chest tube insertion [1,2]. A recent meta-analysis reported pooled rates of 25.9% for pneumothorax and 6.9% for drainage insertion following CLB [1]. While small, asymptomatic pneumothoraces can be managed with observation and supplemental oxygen, larger or rapidly progressive cases can cause significant morbidity and, if left untreated, may be fatal. The standard treatment for the latter is chest tube insertion; however, this intervention carries its own risks, including infection, intercostal nerve injury, and prolonged hospital stays [3].

Several techniques have been proposed to reduce the risk of pneumothorax following CT-guided pulmonary biopsy, including patient positioning strategies, such as rapid roll-over (RR) with the biopsy side down after needle removal or performing the biopsy with the target lung in the dependent position, breathing maneuvers, such as removing the needle during breath-hold, pleural sealing through injection of autologous blood into the pleural space, tract sealing, and various combinations of these approaches [4,5]. At the time of this review, there is no consensus regarding the superiority of any of these techniques, and their use largely depends on operator preference.

This review will further analyze only the effectiveness of tract sealing, which involves the injection of a sealing agent along the biopsy tract during withdrawal of the coaxial guide to prevent post-procedural air leakage. A range of sealing agents has been reported in the literature, including saline solution, autologous blood, hydrogel plug, hemocoagulase, gelatin sponge, gelatin sponge in combination with hemocoagulase, and fibrin glue.

Huo et al. conducted a meta-analysis in 2019 on the effectiveness of these tract sealing agents in preventing pneumothorax and drainage placement; however, only two studies on saline sealing and one study on gelatin sponge were available at that time, and multiple other sealing agents were analyzed together as “tract plugs” [4]. As a substantial number of additional studies have been published since, two more recent meta-analyses have examined the effectiveness of blood patch [6] and gelatin sponge [7] individually, but no contemporary synthesis has investigated all available sealing agents.

The aim of this review and meta-analysis is to evaluate the effectiveness of all tract sealing agents in preventing pneumothorax and drainage insertion after CLB within the same analytical framework.

## 2. Materials and Methods

The protocol of this review was registered in the PROSPERO database (ID: CRD42024608747), available at https://www.crd.york.ac.uk/PROSPERO/view/CRD42024608747 (accessed on 29 January 2026).

This review has been structured according to the Preferred Reporting Items for Systematic reviews and Meta-Analyses (PRISMA 2020) guidelines [8].

**Eligibility criteria**

Randomized controlled trials, as well as prospective and retrospective cohort studies published in English since the databases’ inception, were considered eligible. In accordance with the PICO framework, studies had to meet all of the following criteria:

- Population: Individuals who underwent CLB. Studies reporting CT-guided pulmonary fiducial marker placement were also included, as the procedural technique closely parallels that of biopsy;
- Intervention: Injection of any substance into the lung parenchyma during withdrawal of the coaxial guide, with the purpose of preventing pneumothorax;
- Comparator: Patients in whom no sealing agent was administered following biopsy; patients who received a different sealing agent for pneumothorax prevention;
- Outcomes: pneumothorax and/or pleural drainage insertion.

Exclusion criteria were as follows:

- Conference abstracts, unpublished manuscripts, reviews, case reports, editorials, and comments;
- Studies including fluoroscopy- or ultrasound-guided biopsies;
- Studies including ablation procedures;
- Studies including the pleural patch technique;
- Studies with no comparator group;
- Duplicate or overlapping patient populations.

**Literature search strategy**

Two reviewers (AR and NLT) independently performed database screening and study selection according to the eligibility criteria, as well as data extraction. Discrepancies were discussed by the two reviewers in order to achieve consensus. Four health science databases (ScienceDirect, PubMed, Scopus, and Cochrane Library) were searched up to 13 October 2025. The detailed search strategy can be found in Appendix A. Duplicates were removed by manual verification. Articles assessed for eligibility were organized in EndNote (version 21.0.1) libraries. Full-text reference lists of all included studies were examined to identify additional relevant papers. Corresponding authors of two studies were contacted via e-mail to request information about potential population overlap. Only one author responded, providing clarifications.

**Quality assessment**

The risk of bias was assessed using the *Revised Cochrane risk-of-bias tool for randomized trials (RoB 2)* and *The Risk Of Bias In Non-randomized Studies—of Interventions, Version 2 (ROBINS-I V2)* tool for cohort studies [9,10]. Two authors independently evaluated the risk of bias, and consensus was reached after discussing any discrepancies. The overall risk of bias was equivalent to that of the individual domain with the worst score. In addition, cohort studies with temporally overlapping groups and without control of confounding factors were considered to have an overall critical risk of bias, as the presence of pneumothorax risk factors may have influenced the decision to use tract sealing agents.

**Data extraction**

General study information was extracted as follows: study type, year of publication, number of patients, patient age, tract sealant type, needle and guide gauge, rapid roll-over (RR) technique, presence of emphysema, lesion size, and timing of post-procedural imaging for the detection of pneumothorax. For cohort studies, it was noted whether they included consecutive groups or whether there was temporal overlap between the groups. Missing or unclear information was not recorded.

The outcomes of interest were post-procedural pneumothorax and pleural drainage insertion. Pneumothorax was defined as any air accumulation in the pleural space, regardless of severity, reported by the authors based on post-procedural radiography or CT. When pneumothorax rates were reported at multiple time points, the highest reported rate was used. Pleural drainage insertion was defined as placement of a catheter into the pleural space for treatment of a pneumothorax deemed rapidly expanding or symptomatic by the study authors.

**Statistical analysis**

The two outcomes were analyzed as dichotomous variables (event vs. no event). Effect sizes were expressed as odds ratios (ORs) with corresponding 95% confidence intervals (CIs). Pooled analyses were performed separately for each sealing agent and for each outcome (pneumothorax and drainage insertion) using the Mantel–Haenszel method. Given the anticipated methodological and clinical heterogeneity across studies, random-effects models were used, with between-study variance estimated using the DerSimonian–Laird method. Statistical heterogeneity was quantified using the I^2^ statistic. In the presence of substantial heterogeneity (I^2^ ≥ 50%), sensitivity analyses were conducted by excluding studies identified as major sources of heterogeneity to evaluate their impact on the overall results.

All analyses were performed using Review Manager (RevMan), version 5.4.1. Results were displayed graphically as forest plots for each analysis, ordered by study weight.

If fewer than 10 studies were available for each analysis, we did not conduct funnel plots for publication bias and small-study effects, as these methods are not reliable with small numbers of studies [11]. If at least ten studies were available for an analysis, we performed a visual inspection of the funnel plot to assess potential bias.

Certainty of evidence for each outcome was assessed using the GRADE approach in GRADEpro GDT (McMaster University, 2022 [12]).

## 3. Results

**Literature search and study characteristics**

**Figure 1:**
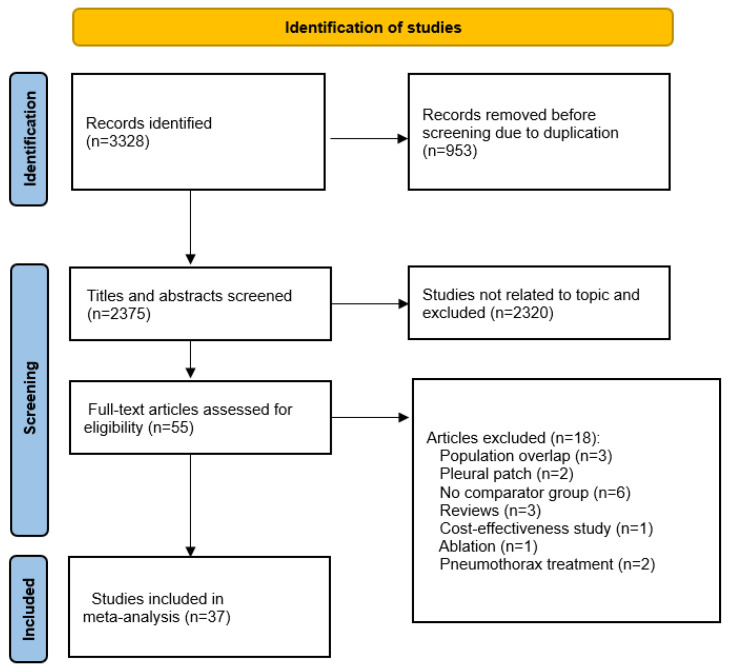
Literature search and study characteristics [8].

A total of 3328 records were initially retrieved through our systematic search. After removal of 953 duplicates, 2375 titles and abstracts were screened, of which 2320 were excluded for irrelevance to the study topic. The remaining 55 articles were evaluated in full text for eligibility. Of these, 18 studies were excluded for the reasons shown in Figure 1. A total of 37 studies, encompassing 13,107 patients (7161 male and 4526 female), met the eligibility criteria and were included in the review. Of these, eight were randomized controlled trials [13,14,15,16,17,18,19,20] and 29 were cohort studies [21,22,23,24,25,26,27,28,29,30,31,32,33,34,35,36,37,38,39,40,41,42,43,44,45,46,47,48,49]. The general study characteristics are shown in Appendix B. Expanded risk-of-bias details are available in Appendix C.

**Figure 2:**
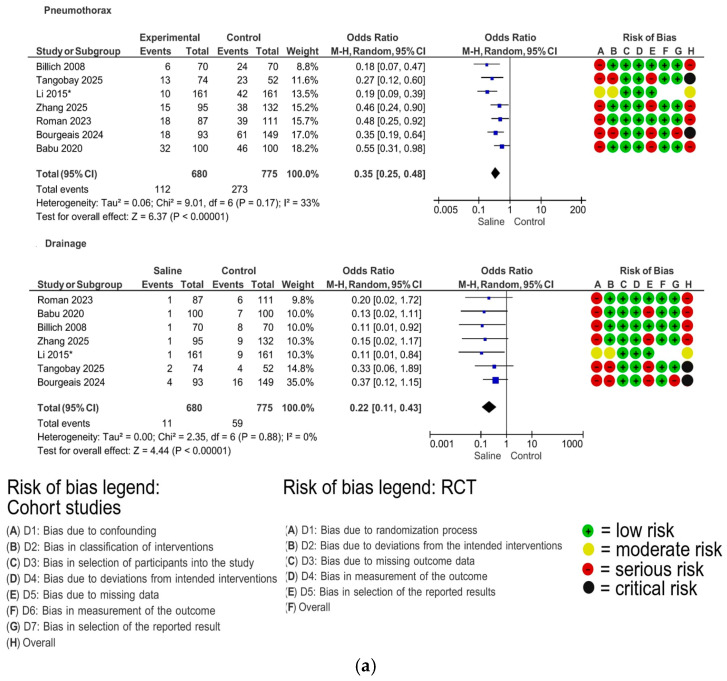

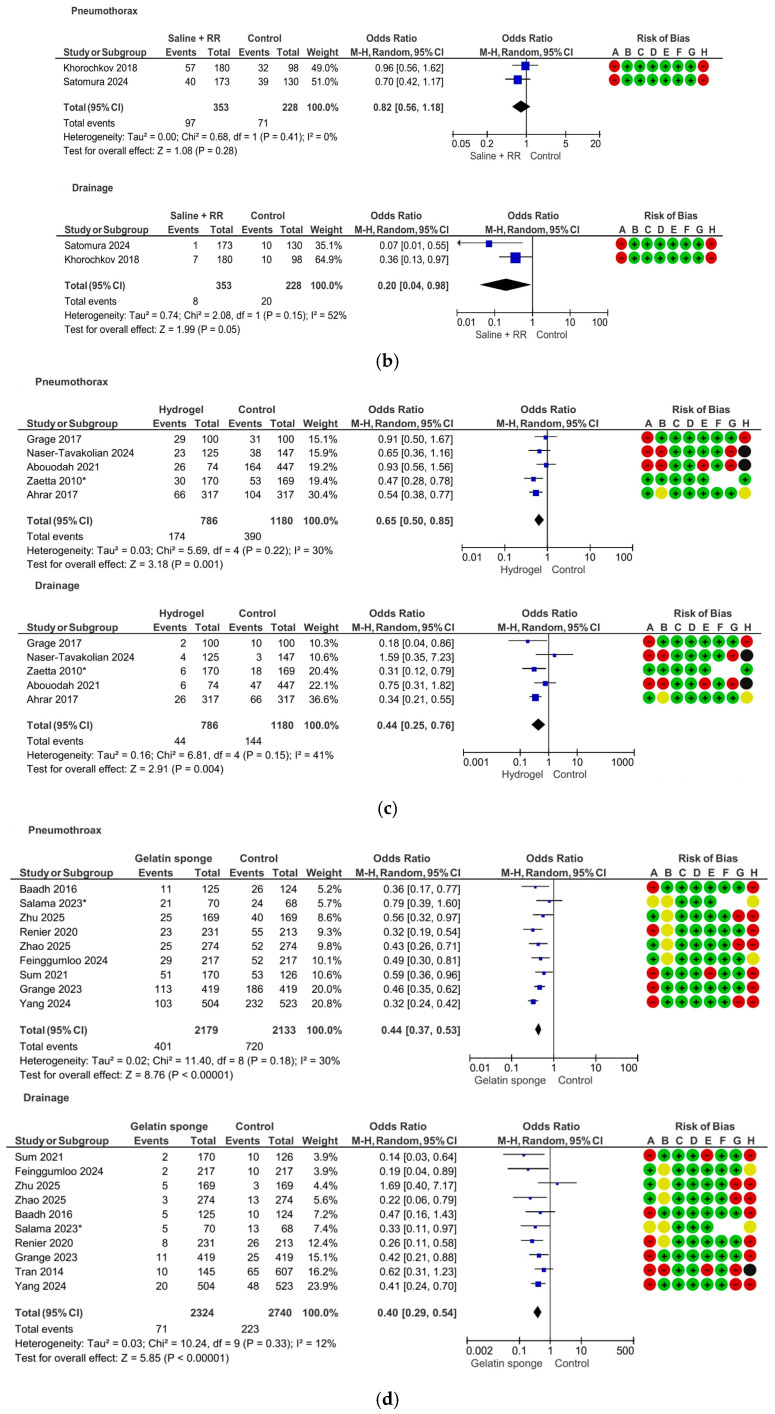

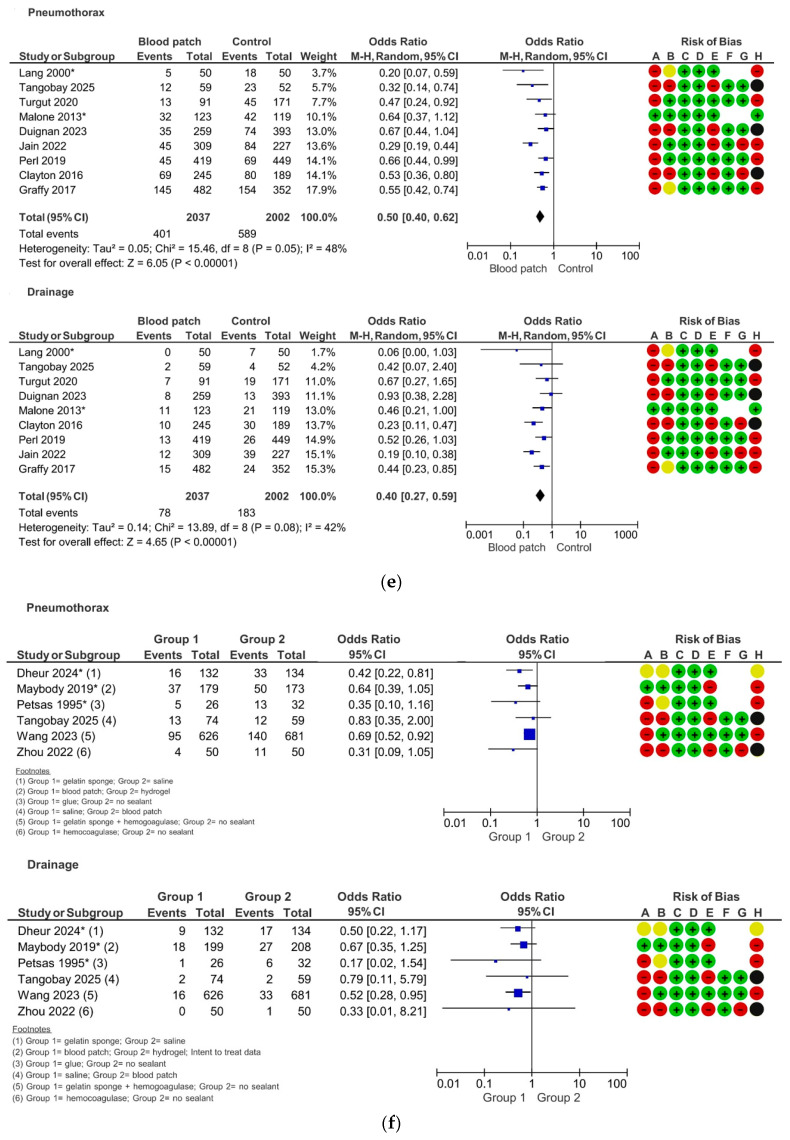
(**a**) Analyses performed for studies using saline solution. (**b**) Analyses performed for studies using saline solution + RR. (**c**) Analyses performed for studies using a hydrogel plug. (**d**) Analyses performed for studies using gelatin sponge. (**e**) Analyses performed for studies using a blood patch. (**f**) Analyses performed for studies using other sealing agents. Randomized controlled trials are indicated by an asterisk; all other studies were cohort studies [13,14,15,16,17,18,19,20,21,22,23,24,25,26,27,28,29,30,31,32,33,34,35,36,37,38,39,40,41,42,43,44,45,46,47,48,49].

The sealing agents reported in the included studies were saline solution, hydrogel plug, gelatin sponge, blood patch, saline solution + rapid roll-over (RR), hemocoagulase, gelatin sponge + hemocoagulase, and fibrin glue. Meta-analyses were performed for sealing agents that were reported in at least two studies using the same type of control group, as follows (Figure 2).

**Saline solution**

Seven studies comparing saline sealing versus no sealant were included, comprising six cohort studies [24,25,26,42,43,49] and one RCT [17]. The use of saline sealing resulted in a significant reduction in pneumothorax (OR = 0.35; 95% CI 0.25–0.48; *p* < 0.00001) and drainage insertion (OR = 0.22; 95% CI 0.11–0.43; *p* < 0.00001). No sensitivity analysis was performed, as heterogeneity was non-substantial for both pneumothorax (I^2^ = 33%) and drainage (I^2^ = 0%) (Figure 2a).

Two cohort saline sealing studies [34,37] were analyzed separately as the intervention group included RR, whereas the control group did not (saline sealing + RR vs. control). The use of saline sealing + RR did not result in a significant reduction in pneumothorax (OR = 0.82; 95% CI 0.56–1.18; *p* = 0.41) but was associated with a reduction in drainage insertion (OR = 0.20; 95% CI 0.04–0.98; *p* = 0.05), approaching statistical significance. Heterogeneity was non-substantial for pneumothorax (I^2^ = 0%) and substantial for drainage (I^2^ = 52%) (Figure 2b).

**Hydrogel plug**

Five studies comparing hydrogel plug sealing versus no sealant were included, comprising four cohort studies [21,22,31,33] and one RCT [16]. The use of a hydrogel plug resulted in a significant reduction in pneumothorax (OR = 0.65; 95% CI 0.50–0.85; *p* = 0.001) and drainage insertion (OR = 0.44; 95% CI 0.25–0.76; *p* < 0.004). No sensitivity analysis was performed, as heterogeneity was non-substantial for both pneumothorax (I^2^ = 30%) and drainage (I^2^ = 41%) (Figure 2c).

**Gelatin sponge**

Nine studies comparing gelatin sponge sealing versus no sealant were included for the pneumothorax outcome, comprising eight cohort studies [23,29,36,38,41,44,46,48] and one RCT [15]. For the drainage outcome, one additional cohort study was included [39]. The use of gelatin sponge resulted in a significant reduction in pneumothorax (OR = 0.44; 95% CI 0.37–0.53; *p* < 0.00001) and drainage insertion (OR = 0.40; 95% CI 0.29–0.54; *p* < 0.00001). No sensitivity analysis was performed, as heterogeneity was non-substantial for both pneumothorax (I^2^ = 30%) and drainage (I^2^ = 12%) (Figure 2d).

**Figure 3:**
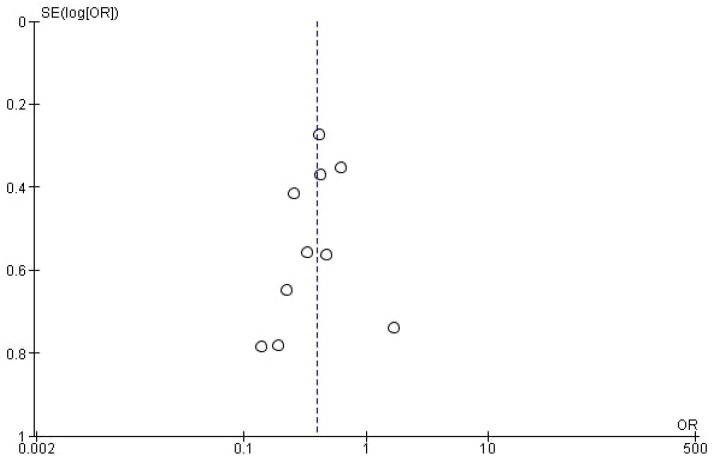
Funnel plot inspection showing no obvious asymmetry suspicious for reporting bias.

As the drainage outcome analysis included ten studies, funnel plot inspection was performed, which showed no obvious asymmetry suspicious for reporting bias (Figure 3).

**Blood patch**

Nine studies comparing blood patch sealing versus no sealant were included, comprising seven cohort studies [27,28,30,32,35,40,49] and two RCT [13,14]. The use of blood patch resulted in a significant reduction in pneumothorax (OR = 0.50; 95% CI 0.40–0.62; *p* < 0.00001) and drainage insertion (OR = 0.40; 95% CI 0.27–0.59; *p* < 0.00001). No sensitivity analysis was performed, as heterogeneity was non-substantial for both pneumothorax (I^2^ = 48%) and drainage (I^2^ = 42%) (Figure 2e).

**Certainty of evidence**

A summary of the results from previous meta-analyses, along with the corresponding certainty of evidence assessments according to the GRADE system, is presented in Table 1.

**Other sealing agents and comparisons**

Three studies directly compared different sealing agents with one another (Figure 2f). Dheur et al. [20] reported that gelatin sponge sealing resulted in a significantly lower pneumothorax rate compared with saline solution (OR = 0.42; 95% CI 0.22–0.81; *p* = 0.008), with no significant difference in drainage insertion. Maybody et al. [19] showed no significant difference between blood patch and hydrogel plug, whereas Tangobay et al. [49] found no significant difference between saline solution and autologous blood patch in preventing either pneumothorax or drainage insertion.

Three studies compared other sealing agents with control groups in which no sealing technique was used. Petsas et al. [18] reported that fibrin glue sealing was associated with a significant reduction in drainage insertion (OR = 0.17; 95% CI 0.02–1.54; *p* = 0.025); the confidence interval crossed unity, which may indicate a statistical or reporting inconsistency. No significant effect was observed for pneumothorax prevention. Wang et al. [45] reported a significant reduction in both pneumothorax (OR = 0.69; 95% CI 0.52–0.92; *p* < 0.001) and drainage insertion (OR = 0.52; 95% CI 0.28–0.95; *p* = 0.002) following tract sealing with a combination of gelatin sponge and hemocoagulase. Zhou et al. [47] reported a reduction in pneumothorax following hemocoagulase sealing (OR = 0.31; 95% CI 0.09–1.05; *p* = 0.04), although this result also bordered on statistical significance, and no significant reduction was observed in drainage insertion.

## 4. Discussion

This meta-analysis comprehensively reviews all reported tract sealing agents under the same methodological framework. The relatively high number of studies available for each agent, combined with structured risk-of-bias assessment using RoB 2 and ROBINS-I and certainty appraisal with GRADE, allows an overview of both effectiveness and the quality of the supporting evidence for each sealing agent. As the results of this review show, the most common sealing agents are saline solution, gelatin sponge, hydrogel, and blood patch.

Saline solution consists of sodium chloride (NaCl) dissolved in water at a concentration of 0.9%, resulting in an isotonic solution with respect to blood and extracellular fluids. Only Satomura et al. [37] described a standardized injection protocol (1 mL per cm of needle tract, with a withdrawal speed of 1 cm/s), whereas all other authors reported volumes between 1 and 10 mL, manually injected according to operator preference.

Desiccated polyethylene glycol hydrogel plugs are, to our knowledge, the only sealing agent specifically designed for the prevention of pneumothorax following percutaneous lung biopsy, and are currently marketed under the BioSentry^®^ Tract Sealant System (Merit Medical Systems, South Jordan, UT, USA) [33]. The desiccated hydrogel plug measures 2.5 cm in length and 0.1 cm in diameter and is deployed using a dedicated delivery system that attaches to the coaxial needle.

Gelatin sponge is a sterile, porous material composed of purified animal-derived gelatin that acts as an effective fluid absorbent, swelling in the process. Gelatin sponge was most commonly administered as a slurry by manually fragmenting sponge material and mixing it with saline, by using powdered sponge to form a paste, or by inserting pre-cut sponge “torpedoes” sized to the coaxial needle. Some authors inject the sealant until resistance is felt, then withdraw the coaxial needle while maintaining the sealant in place with the trocar needle [44]. Others inject the material during coaxial needle withdrawal along the entire biopsy tract [38], or only within the lung parenchyma beneath the pleura [36,48].

The blood patch technique consists of injecting autologous blood through the coaxial needle during its removal. Some authors [27,28,30,35,40,49] administered non-clotted blood [13,14,32], whereas others preferred clotted blood. Among the latter, Lang et al. [13] injected the serum into the deeper portion of the tract and the clot in the superficial segment; Jain et al. [32] injected only the clot; Malone et al. [14] repeatedly transferred the coagulated blood between two syringes in order to fragment the clot before injection. Similar to the saline sealing technique, none of the authors reported a standardized injection protocol, mentioning only the approximate volume of blood (ranging between 1 and 8 mL) that was injected upon needle removal. Most authors aimed to fill the entire biopsy tract, while others [14,28] restricted injection to the subpleural lung parenchyma.

The results of our review show that all sealing agents included in the meta-analyses led to a statistically significant reduction in both pneumothorax (OR = 0.35–0.65) and drainage (OR = 0.22–0.44) risk, with the exception of saline sealing + RR, for which only two studies were available (Table 1). Overall, saline sealing appears to provide the most consistent reduction in both pneumothorax and drainage insertion risk, the latter supported by the saline + RR results. A possible explanation is that saline solution disperses into the adjacent airspaces, creating a larger sealant volume that must be displaced before air leakage can occur. Moreover, due to its low viscosity, even if the saline is expelled from an airway during expiration, it could leak back in during inspiration, helping to maintain occlusion. In contrast, hydrogel plugs and gelatin sponge are highly viscous and remain confined to the needle tract, without extending into surrounding airspaces. For this reason, an air leakage might be more likely to form and to persist around the plug. Autologous blood patch, though initially fluid, coagulates rapidly after injection, behaving similarly to viscous agents and potentially leading to similar outcomes.

We have identified only two studies directly comparing two different sealing agents, both of which are RCTs. Maybody et al. concluded that autologous blood patch is non-inferior to hydrogel plug in the prevention of pneumothorax, which is in line with the findings of this review [19]. However, Dheur et al. have shown a significant reduction in pneumothorax rates and a non-significant reduction in drainage rates in favor of gelatin sponge compared to saline solution [20]. This comes in contrast with the findings of our meta-analysis and might be explained by the fact that the authors injected saline only in the superficial 1–2 cm of the lung as opposed to all other reports, where saline was injected along the entire needle tract.

The majority of studies included in this review were cohort studies, many of which shared recurring structural limitations with a high potential for bias. Only a minority implemented methods to control for confounding factors known to influence pneumothorax risk—and consequently, the need for drainage—such as underlying emphysema, needle caliber, patient position, and lesion size or depth [1]. The remaining cohort studies comprised either consecutive patient series with no reported temporal overlap between groups or patient series in which the use of a sealing agent was determined by the operator. In the first scenario, the control group generally consisted of a historical series of patients in whom no sealing method was employed, compared with a more recent series in which sealing was applied to all patients following a departmental decision. This structure provides a degree of pseudo-randomization but introduces other confounding elements, such as increased operator experience over time and the evolving complexity of attempted procedures. In the second scenario, both groups were drawn from the same time period, with operators choosing whether or not to use a sealing agent based on criteria that were often not specified, including personal preference. Studies adopting this approach were judged to be at critical risk of bias, since it is reasonable to assume that operators were more likely to apply a sealing technique in patients perceived to be at higher risk for pneumothorax. However, this bias likely favors the control (non-sealant) group, thereby attenuating the observed effect of the sealing agent.

Another recurring issue was bias due to missing data, as some authors excluded patients who developed pneumothorax prior to the removal of the coaxial needle/application of the sealant. This exclusion is likely problematic, since these patients might have benefited from the sealing procedure, which could have prevented further pneumothorax expansion. This approach is likely related to the authors’ definition of pneumothorax. There was considerable heterogeneity between the included studies regarding both the modality and the timing of pneumothorax assessment. Some authors reported pneumothorax only on the immediate post-procedural CT (which we considered to represent bias in the selection of the reported result), while others defined it based on a chest radiograph performed at a prespecified time after the procedure, or on a combination of the two. We consider the approach of Malone et al. to be the most valid as it counts only the clinically significant events: “A patient was counted as having developed a pneumothorax if any measurable pneumothorax was measured on the 1-h, 3-h, or subsequent radiograph. Some patients developed a pneumothorax that was visible on CT images obtained during the procedure. These patients were counted as having a pneumothorax only if the pneumothorax was detectable on subsequent postprocedural radiographs [14]”.

A potential, though more difficult to quantify, source of bias is the threshold for drainage insertion used by different authors. Terms such as rapidly progressing pneumothorax or symptomatic pneumothorax are inherently vague and open to variable interpretation. These differing thresholds may partly explain the variability in the pooled absolute rates of drainage insertion, which ranged from 7.6% in the saline control group to 12.2% in the hydrogel control group. However, since the clinical decision-making process is likely to have been consistent within each individual study, the odds ratio-based analyses probably compensate for this variability to some extent. Nevertheless, these findings highlight the need for both procedural and follow-up standardization.

A limitation of this study is the absence of meta-regression and, except for the gelatin sponge subgroup, the inability to formally assess publication bias. This reflects the limited number of studies available for most sealing agents, which would render these analyses underpowered and potentially misleading. The absence of meta-regression limits the ability to formally examine whether study-level characteristics modified treatment effects or explained between-study heterogeneity. Consequently, variation related to differences in patient populations, procedural techniques, or study design may remain unaccounted for.

Finally, we note that saline sealing is implemented as standard practice at our institution for pneumothorax prevention [43]. Nonetheless, strict methodological rigor was applied to ensure an unbiased and objective assessment of all sealing techniques included in this review.

## 5. Conclusions

Saline solution, hydrogel plug, gelatin sponge, and autologous blood patch are sealing agents effective at lowering the risk of pneumothorax and drainage insertion following CLB and may be considered as part of standard practice. Although saline sealing demonstrated the largest effect estimates in this review, head-to-head comparative data remain sparse, and no definitive conclusion can be drawn regarding the superiority of any single sealing agent. At present, the choice of sealing technique should be guided by operator familiarity, procedural workflow, resource availability, and patient characteristics. Well-designed, adequately powered randomized controlled trials directly comparing different sealing methods are needed to clarify their relative effectiveness and to support standardization in clinical practice.

## Figures and Tables

**Table 1:** Summary of findings and certainty of evidence assessment according to the GRADE system.

Analysis	Certainty Assessment							No. of Patients		Effect		Certainty
	No. of Studies	Study Design	Risk of Bias	Inconsistency	Indirectness	Imprecision	Other Considerations	Sealant	Control	Relative (95% CI)	Absolute (95% CI)	
Saline: PTX	7	6 cohort 1 RCT	serious	not serious	not serious	not serious	strong association	112/680 (16.5%)	273/775 (35.2%)	**OR 0.35** (0.25 to 0.48)	**192 fewer per 1000** (from 233 fewer to 145 fewer)	⨁⨁⨁⨁ High
Saline: Drainage	7	6 cohort 1 RCT	serious	not serious	not serious	serious	strong association	11/680 (1.6%)	59/775 (7.6%)	**OR 0.22** (0.11 to 0.43)	**58 fewer per 1000** (from 67 fewer to 42 fewer)	⨁⨁⨁◯ Moderate
Saline+RR: PTX	2	2 cohort	serious	not serious	not serious	very serious	none	97/353 (27.5%)	71/228 (31.1%)	**OR 0.82** (0.56 to 1.18)	**41 fewer per 1000** (from 109 fewer to 37 more)	⨁◯◯◯ Very low
Saline+RR: Drainge	2	2 cohort	serious	not serious	not serious	serious	strong association	8/353 (2.3%)	20/228 (8.8%)	**OR 0.20** (0.04 to 0.98)	**69 fewer per 1000** (from 84 fewer to 2 fewer)	⨁⨁⨁◯ Moderate
Hydrogel: PTX	5	4 cohort 1 RCT	very serious	not serious	not serious	not serious	none	174/786 (22.1%)	390/1180 (33.1%)	**OR 0.65** (0.50 to 0.85)	**88 fewer per 1000** (from 133 fewer to 35 fewer)	⨁⨁◯◯ Low
Hydrogel: Drainage	5	4 cohort 1 RCT	very serious	not serious	not serious	serious	strong association	44/786 (5.6%)	144/1180 (12.2%)	**OR 0.44** (0.25 to 0.76)	**64 fewer per 1000** (from 88 fewer to 26 fewer)	⨁⨁◯◯ Low
Gelatin sponge: PTX	9	8 cohort 1 RCT	serious	not serious	not serious	not serious	none	401/2179 (18.4%)	720/2133 (33.8%)	**OR 0.44** (0.37 to 0.53)	**154 fewer per 1000** (from 179 fewer to 125 fewer)	⨁⨁⨁◯ Moderate
Gelatin sponge: Drainage	10	9 cohort 1 RCT	serious	not serious	not serious	not serious	strong association	71/2324 (3.1%)	223/2740 (8.1%)	**OR 0.40** (0.29 to 0.54)	**47 fewer per 1000** (from 56 fewer to 36 fewer)	⨁⨁⨁⨁ High
Blood patch: PTX	9	7 cohort 2 RCT	very serious	not serious	not serious	not serious	none	401/2037 (19.7%)	589/2002 (29.4%)	**OR 0.50** (0.40 to 0.62)	**122 fewer per 1000** (from 151 fewer to 89 fewer)	⨁⨁◯◯ Low
Blood patch: Drainage	9	7 cohort 2 RCT	very serious	not serious	not serious	not serious	strong association	78/2037 (3.8%)	183/2002 (9.1%)	**OR 0.40** (0.27 to 0.59)	**53 fewer per 1000** (from 65 fewer to 35 fewer)	⨁⨁⨁◯ Moderate

Abbreviations: CI = confidence interval; OR = odds ratio; PTX = pneumothorax; RCT = randomized control trial; RR = rapid-rollover.

## Data Availability

The original contributions presented in this study are included in the article. Further inquiries can be directed to the corresponding author.

## Abbreviations

The following abbreviations are used in this manuscript:

CLB	CT-guided lung biopsy.
OR	Odds ratio.
CI	Confidence interval.
RR	Rapid roll-over.

## Appendix A. Search Strategy

- **ScienceDirect (via Elsevier)**—multiple keyword combinations were used to run separate searches for each biological agent:
  − (“Pneumothorax” OR “Complication” OR “Complications” OR “Drainage” OR “Tube”) AND “Biopsy” AND (“Lung” OR “Pulmonary”) AND “Blood Patch”
  − (“Pneumothorax” OR “Complication” OR “Complications” OR “Drainage” OR “Tube”) AND “Biopsy” AND (“Lung” OR “Pulmonary”) AND “Saline”
  − (“Pneumothorax” OR “Complication” OR “Complications” OR “Drainage” OR “Tube”) AND “Biopsy” AND (“Lung” OR “Pulmonary”) AND “Gelfoam”
  − (“Pneumothorax” OR “Complication” OR “Complications” OR “Drainage” OR “Tube”) AND “Biopsy” AND (“Lung” OR “Pulmonary”) AND “Hydrogel”
  − (“Pneumothorax” OR “Complication” OR “Complications” OR “Drainage” OR “Tube”) AND “Biopsy” AND (“Lung” OR “Pulmonary”) AND “Gelatin”
  − (“Pneumothorax” OR “Complication” OR “Complications” OR “Drainage” OR “Tube”) AND “Biopsy” AND (“Lung” OR “Pulmonary”) AND “Plug”
  − (“Pneumothorax” OR “Complication” OR “Complications” OR “Drainage” OR “Tube”) AND “Biopsy” AND (“Lung” OR “Pulmonary”) AND “Biosentry”
  − (“Pneumothorax” OR “Complication” OR “Complications” OR “Drainage” OR “Tube”) AND “Biopsy” AND (“Lung” OR “Pulmonary”) AND “Collagen”
  − (“Pneumothorax” OR “Complication” OR “Complications” OR “Drainage” OR “Tube”) AND “Biopsy” AND (“Lung” OR “Pulmonary”) AND “Glue”
- **Scopus (via Elsevier)**—(TITLE-ABS-KEY (“Pneumothorax” OR “Complication” OR “Complications” OR “Drainage” OR “Tube”) AND TITLE-ABS-KEY (“Biopsy” OR “Intervention”) AND TITLE-ABS-KEY (“Lung” OR “Pulmonary”) AND TITLE-ABS-KEY (“Blood Patch” OR “Saline” OR “Gelfoam” OR “Hydrogel” OR “Gelatin” OR “Plug” OR “Sealing” OR “Biosentry” OR “Foam” OR “Polymer” OR “Composite” OR “Composites” OR “Glue” OR “Collagen”) AND (LIMIT-TO (LANGUAGE, “English”)) AND (LIMIT-TO (EXACTKEYWORD, “Observational Study”) OR LIMIT-TO (EXACTKEYWORD, “Randomized Controlled Trial (topic)”) OR LIMIT-TO (EXACTKEYWORD, “Cohort Analysis”) OR LIMIT-TO (EXACTKEYWORD, “Controlled Study”) OR LIMIT-TO (EXACTKEYWORD, “Follow-Up Studies”) OR LIMIT-TO (EXACTKEYWORD, “Prospective Studies”) OR LIMIT-TO (EXACTKEYWORD, “Prospective Study”) OR LIMIT-TO (EXACTKEYWORD, “Randomized Controlled Trial”) OR LIMIT-TO (EXACTKEYWORD, “Retrospective Studies”) OR LIMIT-TO (EXACTKEYWORD, “Retrospective Study”))
- **Cochrane Library (via Wiley)**—(“Pneumothorax” OR “Complication” OR “Complications” OR “Drainage” OR “Tube”) AND (“Biopsy” OR “Intervention”) AND (“Lung” OR “Pulmonary”) AND (“Blood Patch” OR “Saline” OR “Gelfoam” OR “Hydrogel” OR “Gelatin” OR “Plug” OR “Sealing” OR “Biosentry” OR “Foam” OR “Polymer” OR “Composite” OR “Composites” OR “Glue” OR “Collagen”)
- **Pubmed (via NCBI)**—(“Pneumothorax” OR “Complication” OR “Complications” OR “Drainage” OR “Tube”) AND (“Biopsy” OR “Intervention”) AND (“Lung” OR “Pulmonary”) AND (“Blood Patch” OR “Saline” OR “Gelfoam” OR “Hydrogel” OR “Gelatin” OR “Plug” OR “Sealing” OR “Biosentry” OR “Foam” OR “Polymer” OR “Composite” OR “Composites” OR “Glue” OR “Collagen”).

## Appendix B

**Table A1:** General study characteristics.

Author	Year	Conesecutive—Only to Cohort Studies	Study Design	Sealant	Country	Rapid Roll-Over		Number of Patients		Male/Female (n)		Age (y)—Mean		Emphysema		Lesion Size (mm)—Mean		Guide and Biopsy Needle Gauge	Diagnosis of Pneumothorax (h)
						Sealant	Control	Sealant	Control	Sealant	Control	Sealant	Control	Sealant	Control	Sealant	Control		
Babu [24]	2020	Y	cohort	saline (1–3 mL)	Singapore	no	no	100	100	-	-	64.8	68.3	16	18	39	37	16/18G or 19/20G	0 h, 4 h
Li [17]	2015	-	RCT	saline (1–3 mL)	China	no	no	161	161	107/54	111/50	57.3	58.2	41	37	32	33	19/20G	0 h, 6 h
Billich [25]	2008	N	cohort	saline (2–4 mL)	Germany	no	no	70	70	52/18	49/21	63.64	62.76	3	1	30.1	29.4	16/18G	0 h, 2 h
Bourgeais [26]	2024	N	cohort	saline (3–5 mL)	France	no	no	93	149	64/29	97/52	70	69	51	82	30	25	17/18G or 19/20G	0 h
Zhang [42]	2025	Y	cohort	saline (3–5 mL)	China	no	no	95	132	52/43	81/51	65.7	67.5	2	3	28.3	29.5	17/18G	0 h, 4 h
Satomura [37]	2024	Y	cohort	saline (1–5 mL)	Japan	yes	no	173	130	105/68	84/46	71.5	69.7	80	61	19.2	25.7	17/18G	0 h, 2 h
Tangobay [49]	2025	N	cohort	saline (5–10 mL)	Turkey	no	no	74	52	143/42		64.55		-	-	32		17/18G or 19/20G or 22G	0 h, 4 h
Khorochkov [34]	2018	Y	cohort	saline (3–5 mL)	Canada	yes	no	180	98	82/98	39/59	71	68	-	-	-	-	19/20G	2 h
Roman [43]	2023	Y	cohort	saline (3–5 mL)	Romania	no	no	87	111	40/47	69/42	63.4	63.7	31	31	29.2	32.7	17/18G	0 h, 2 h
Grage [31]	2017	Y	cohort	hydrogel plug	USA	no	no	100	100	46/54	57/43	66.92	62.92	83	86	19.7	23.3	19/20G	0 h, 2 h
Zaetta [16]	2010	-	RCT	hydrogel plug (BioSeal)	USA	no	no	170	169	78/92	94/75	66.1	67.2	-	-	under 30		19G	0 h, 24 h, 30 day
Aboudodah [21]	2021	N	cohort	hydrogel plug (Biosentry)	USA	no	no	74	447	36/38	234/213	67.3	65.4	1	3	-	-	-	0 h, 24 h
Ahrar [22]	2017	N	cohort	hydrogel plug (Biosentry)	USA	no	no	317	317	156/161	165/152	63.3	63.4	83	87	19.8	21.2	19/20G or 19/22G	0 h, 3 h
Naser-Tavakolian [33]	2024	N	cohort	hydrogel plug (Biosentry)	USA	no	no	125	147	38/87	69/78	70.05	68.95	59	44	19.2	26.3	17/18G	0 h, 1 h
Renier [36]	2020	Y	cohort	gelatin sponge (Gelfoam)	Belgium	yes	yes	231	213	129/102	110/103	65.7	65.1	46	43	23.8	25.6	19/20G	0 h, 4 h
Sum [38]	2021	Y	cohort	gelatin sponge (Gelfoam)	Australia	no	no	170	126	67/59	94/76	69	71	55	42	-	-	19/20G	0 h, 4 h
Yang [41]	2024	Y	cohort	gelatin sponge (Gelfoam)	China	no	no	504	523	359/145	170/153	68.21	67.72	212	219	30.1	31.51	18/20G	0 h
Salama [15]	2023	-	RCT	gelatin sponge (Gelfoam)	Egypt	no	no	70	68	55/15	50/18	60.3	55.2	32	27	-	-	17/18G	0 h, 1 h, 3 h
Zhao [46]	2025	N	cohort	gelatin sponge (Gelfoam)	China	no	no	274	274	137/137	136/138	62.19	62.26	56	59	44.14	44.82	17/18G	0 h
Zhu [48]	2025	N	cohort	gelatin sponge (Gelfoam)	China	no	no	169	169	107/62	112/57	66.9	67.3	62	58	32	32	17/18G	0 h
Grange [44]	2023	N	cohort	gelatin sponge (Gelitaspon)	France	no	no	419	419	63/42	65/35	66.2	66.7	191	174	25.6	26.6	18G or 19G or 20G	0 h, 4 h
Wang [45]	2023	Y	cohort	gelatin sponge (Jiangxi Xiangen Medical Technology Devel- opment Co.) + haemocoagulase	China	no	no	626	681	331/295	389/292	63.28	63	-	-	33.67	34.9	17/18G	0 h, 3 h
Feinggumloon [29]	2024	N	cohort	gelatin sponge (Spongostan)	Thailand	no	no	217	217	97/120	97/120	64.9	64.4	12	11	32.9	28.9	16G or 17G	0 h, 4 h
Baadh [23]	2016	Y	cohort	gelatin sponge (Surgifoam)	USA	no	no	125	124	49/76	66/58	72.4	79.4	-	-	29	30	17/18G or 19/20G	0 h, 1 h
Tran [39]	2014	N	cohort	gelatin sponge (Surgifoam)	USA	no	no	145	607	81/64	304/303	68.26	63,51	19	66	30.74	28.86	19/21G or19/20G	0 h, 4 h, 6 h
Jain [32]	2022	Y	cohort	ABP clotted	USA	no	no	309	227	146/163	107/120	69	68	-	-	-	-	19/20G or 17/20G	0 h
Lang [13]	2000	-	RCT	ABP clotted	USA	no	no	50	50	63/37		54	48	21	17	20	24	19G	0 h, 4 h
Malone [14]	2013	-	RCT	ABP clotted	USA	no	no	123	119	52/71	60/59	65	66	34	37	2.2	2.3	17/18G or 19/20G	1 h, 3 h
Clayton [27]	2016	Y	cohort	ABP nonclotted	USA	no	no	245	189	125/64	147/98	67	66	115	93	23		19/20G or 19/22G	0 h, 2 h
Duignan [28]	2023	N	cohort	ABP nonclotted	Ireland	yes	yes	259	393	109/150	184/209	68.4	68.1	48	21	24	28	19/20G	1 h, 4 h
Graffy [30]	2017	Y	cohort	ABP nonclotted	USA	yes	yes	482	352	278/204	206/146	66.2	63.4	200	146	-	-	19/20G or 19/21G	1 h
Perl [35]	2019	Y	cohort	ABP nonclotted	Germany	no	no	419	449	-	-	-	-	-	-	-	-	13G or 15G or 17G or 19G	2 h
Tangobay [49]	2025	N	cohort	ABP nonclotted	Turkey	no	no	59	52	143/42		64.55		-	-	32		17/18G or 19/20G or 22G	0 h, 4 h
Turgut [40]	2020	Y	cohort	ABP nonclotted	Turkey	no	no	91	171	80/11	153/18	62.6	59.3	8	12	32	30	19/20G	0 h, 6 h
Petsas [18]	1995	-	RCT	fibrin glue (Tissucol 0.5)	Greece	yes	yes	26	32	41/17		-	-	-	-	-	-	19G/22G	0 h, 1 h, 4 h, 24 h
Zhou [47]	2022	N	cohort	haemocoagulase	China	no	no	50	50	44/56		60	64	-	-	27	28	17/18G	0 h
**Studies comparing two different sealing agents**								**Sealant no.1**	**Sealant no.2**	**Sealant no.1**	**Sealant no.2**	**Sealant no.1**	**Sealant no.2**	**Sealant no.1**	**Sealant no.2**	**Sealant no.1**	**Sealant no.2**		
Maybody [19]	2019	-	RCT	ABP (no.1) vs. hydrogel - Biosentry (no.2)	USA	no	no	179	173	-	-	-	-	-	-	-	-	-	2 h
Dheur [20]	2024	-	RCT	saline (no.1) vs. gelatin sponge (no.2)	France	no	no	134	132	78/56	72/60	65.7	67.2	76	67	18	18	19/20G	0 h, 4 h

Y = yes; N = no; RCT = randomized controlled trial; h = hours; n = number; y = years; G = Gauge.

## Appendix C. Risk of Bias

**Saline solution**

Two cohort studies [26,49] were assessed as being at critical risk of bias, as the use of the sealing agent was determined by the operator’s choice, with no control for confounding factors. The remaining cohort studies, consisting of consecutive patient series with no reported temporal overlap between groups, were assessed as being at serious risk of bias due to uncontrolled confounding factors and, in two studies [24,42], missing outcome data from excluded patients. The RCT was judged to have a moderate risk of bias, primarily due to flaws in the randomization process and deviations from the intended interventions.

**Hydrogel plug**

Two cohort studies [21,33] were assessed as being at critical risk of bias, as the use of the sealing agent was determined by the operator’s choice, with no control for confounding factors. One cohort study [31], consisting of consecutive patient series with no reported temporal overlap between groups, was assessed as being at serious risk of bias due to uncontrolled confounding factors. One propensity-matched cohort study [22] was assessed as being at moderate risk of bias, as the use of the sealing agent was determined by the operator’s choice. The RCT [16] was judged to have a low risk of bias.

**Gelatin sponge**

One cohort study [39] was assessed as being at critical risk of bias, as the use of the sealing agent was determined by the operator’s choice, with no control for confounding factors. Five cohort studies [23,36,38,41,44], consisting of consecutive patient series with no reported temporal overlap between groups, were assessed as being at serious risk of bias due to uncontrolled confounding factors, as well as missing outcome data from excluded patients [38] and had an incomplete evaluation of the pneumothorax rate, as it was assessed only on the post-procedural CT [41]. Of the three propensity-matched cohorts, two [46,48] were assessed as being at serious risk of bias due to incomplete evaluation of the pneumothorax rate, as it was assessed only on the post-procedural CT, and one [29] as being at moderate risk of bias, as the use of the sealing agent was determined by the operator’s choice. The RCT [15] was judged to have a moderate risk of bias, primarily due to flaws in the randomization process and deviations from the intended interventions.

**Blood patch**

Three [27,28,49] cohort studies were assessed as being at critical risk of bias, as the use of the sealing agent was determined by the operator’s choice, with no control for confounding factors. The remaining cohort studies, consisting of consecutive patient series with no reported temporal overlap between groups, were assessed as being at serious risk of bias due to uncontrolled confounding factors, and in one study [32], there was missing outcome data from excluded patients, as well as incomplete evaluation of the pneumothorax rate, as it was assessed only on the post-procedural CT. One RCT [13] was judged to have a serious risk of bias, primarily due to flaws in the randomization process and deviations from the intended interventions, while the other [14] had a low risk of bias.

**Saline solution + RR**

Both studies, consisting of consecutive patient series with no reported temporal overlap between groups, were assessed as being at serious risk of bias due to uncontrolled confounding factors [34,37].
